# Perspective isomorphs – a new classification of molecular structures based on artistic and chemical concepts

**DOI:** 10.3762/bjoc.15.224

**Published:** 2019-09-30

**Authors:** Jannis Neumann, Ansgar Schnurr, Hermann A Wegner

**Affiliations:** 1 Justus-Liebig-Universität Gießen, Institut für Kunstpädagogik, Karl-Glöckner-Str. 21, 35394 Gießen, Germany; 2 Justus-Liebig-Universität Gießen, Institut für Organische Chemie, Heinrich-Buff-Ring 17, 35392 Gießen

**Keywords:** art and science, nomenclature, perspective, representation in chemistry, structure

## Abstract

Models are a quintessential part in research as well as for scientific communication in general. A special challenge is the visualization of the invisible, such as atoms and molecules. Visualizations are also deeply rooted in the discipline of art offering in the respect untapped potential in cross-fertilization with natural sciences. Here we show a new classification of molecular structures, so-called perspective isomorphs, applying an interdisciplinary crossing of epistemological concepts between chemistry and art. The idea is based on the notion that molecules can be classified, if they appear equivalent from one standpoint in a specific orientation. We termed such a group of such molecules perspective isomorphs. The general concept is outlined together with a nomenclature system. Furthermore, this concept has been visualized by artistic representations of molecules. The concept of perspective isomorphs and its discussions herein will extend current models and stimulate the discourse about the nature of atoms and molecules and especially their models.

## Introduction

Up to now, epistemological concepts of molecules as visualized in chemical models are marked by an omniscient, all-seeing understanding [1]. The methods of representing/depicting molecules (in various kinds of formulae and projections) include neither the human viewer nor their point of view. The spatial dimension in this understanding is rather restricted to the scale of a molecule and the directionality of its interactions with other molecules. Nevertheless, the three-dimensional structure of a molecule is decisive for its characteristic properties and hence its function in biological systems and materials [2].

In contrast, artistic concepts of visually based cognition from the renaissance to modern age derive predominately from the human act of observing. The visually captured world is perceived as a spatially arranged scene in perspective dependency to the viewer. For pictorial representation, only the visible is relevant. Due to the size of molecules (sub-nm) such direct observations of chemical compounds by the human eye are not possible. This results in a different understanding of the spatial relations of these objects and the observer. Because a molecule’s interaction with its environment differs depending on its position and orientation, we suggest to include the molecule’s directionality in space and, an imaginary observer’s specific point of view.

Herein, we show an approach implementing a new classification of molecules, which derives from the artistic concept of visual perception and perspective relationships. From this cross-disciplinary transfer we propose the term *perspective isomorphs*, describing molecules that appear identical from a certain viewpoint, but differ structurally. Furthermore, we aim at triggering a new discourse in chemical research on the epistemological dimension of visualizations. To initiate this discourse and to find innovative model notations, contemporary artists were asked to find new graphic ways of representing atoms.

The methodical approach is not in the actual seeing, but rather the conceptualization of an epistemology using parameters, which are derived from the human visual ability. The access of our findings is marked by a viewpoint, a viewing angle, and the human spatial perception. Thus, the act of perceiving a visual image of molecules will be called vision in this paper, due to the lack of a better term and the presupposition of this act being hypothetical.

## Results and Discussion

### Contextualization

The understanding of space in chemistry is highly linked to the three-dimensional extensions of molecules and their directionality in chemical bonds, reactions, and processes. In this context, interactions, such as hydrogen-bonds [3], metal-coordination [4] or quadrupole interactions [5–6] between molecules or specific parts within molecules, are highly dependent on the orientation of the interacting entities. In contrast, considerations of space on a supramolecular scale [7–8] or even the integration of an actual human viewer are mostly excluded in models of chemical concepts. The research method of observing visual phenomena is naturally bound to the physiological capabilities of the visual apparatus and the processing brain structures. However, these methods have constantly been enhanced by the invention and improvement of scientific instruments [9–10]. Other than only focusing on the observed object, we suggest to enhance the method further by understanding the act of observation as an active process, which requires the integration of the observer with his distinct point of view. Thus, space is not only understood in intermolecular dimensions, but in an organismic relation including the organism’s specifications and limitations in the visual experience of space.

The invention of the central perspective as a spatial and pictorial concept in the Renaissance closely associated the human being´s physiological visual impression with the epistemological process of cognition. Later artistic concepts developed alternative concepts of decentered perspective. On the basis of mathematical and visual considerations of Alhazen’s Arabian theory of vision in the eleventh century [11], European artists of the early modern age transformed Arabian thought into western art by conceptualizing their physical (one-eyed) visual impression and projected the perception of parallel and converging lines and staggering forms onto the canvas [12]. Two aspects are significant here: First of all, items on a canvas appear radically in the way they are visually apprehensible (backsides and concealments are invisible; sizes depend on the distance to the subject); furthermore, the picture is formed only by the individual location of the person. Pictorial presentation is no longer characterized through a metaphysical interpretation or an abstract, all-knowing concept, but the result of the act of observing [13]. From this viewpoint, this means the conception of the world is formed by the sensory experience of the individual, which is highly bound to the spatial position of the experiencing one, especially in case of the visual sense.

Because the cognitive achievement radically corresponds to the possibilities of the perceiving subject – recognizing only what is perceptible – the invention of the central perspective can be interpreted as a pre-history of today's discourse on radical constructivism [14]. Based on interdisciplinary studies, including natural science, neuroscience, psychology, and philosophy radical constructivism declares exclusively the sensible world, as it can be recognized by our sensory organs, as epistemological basis of all world knowledge.

This positional relationship between the viewer and an object can be conceptually transferred onto two molecular entities revealing the possibility of specific orientation and space-depended interactions, such as hydrogen-bonds, etc., which is of utmost importance for instance in any biological process [15].

### Proposal of perspective isomorphs

When applying the principle of the central perspective to the view on molecules, their representation is governed by the perspective of a real or imaginative eye. Thus, different molecules can appear the same if a specific perspective is chosen. We propose the term *‘perspective isomorphs’* for such a class of molecules*.* Therefore, a class of perspective isomorphs (PI class in short) is defined as a group of molecules that are congruent in a hypothetical view with the human eye and from a defined perspective. Thus, the spatial organization of their incorporated atoms would not be distinguishable for the human mode of perception. Such molecules are called *perspective isomorphs* of a specific PI class. The challenges when modeling scientific concepts are that they can never be more than an approximation in the construction of a reality, in order to pursue its representation. Therefore, certain assumptions have to be made beforehand and are outlined in the following guidelines, which define the hypothetical view of the molecules by the human eye:

- Atoms are considered with a spherical shape with a high density in the center, gradually decreasing to the outward
- The individual atoms of a perspective isomorph have no gaps or overlaps with each other, but touch directly at their peripheral borders (akin to van der Waals contacts)
- All conformations possible at room temperature are considered
- To translate the atomic dimensions to a viewing process the van der Waals radii and the following scale has been applied: 1 pm 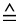 1 mm
- The hypothetical viewing distance for the mathematic calculation as well as for the artistic presentation is determined as 2250 mm from the eye to the center of the first atom of the perspective isomorph. On this scale the perspective shortening can be neglected.

In the examples described in this report only the elements hydrogen, carbon, nitrogen, oxygen, and fluorine have been taken into consideration to illustrate the general concept.

**Figure 1:**
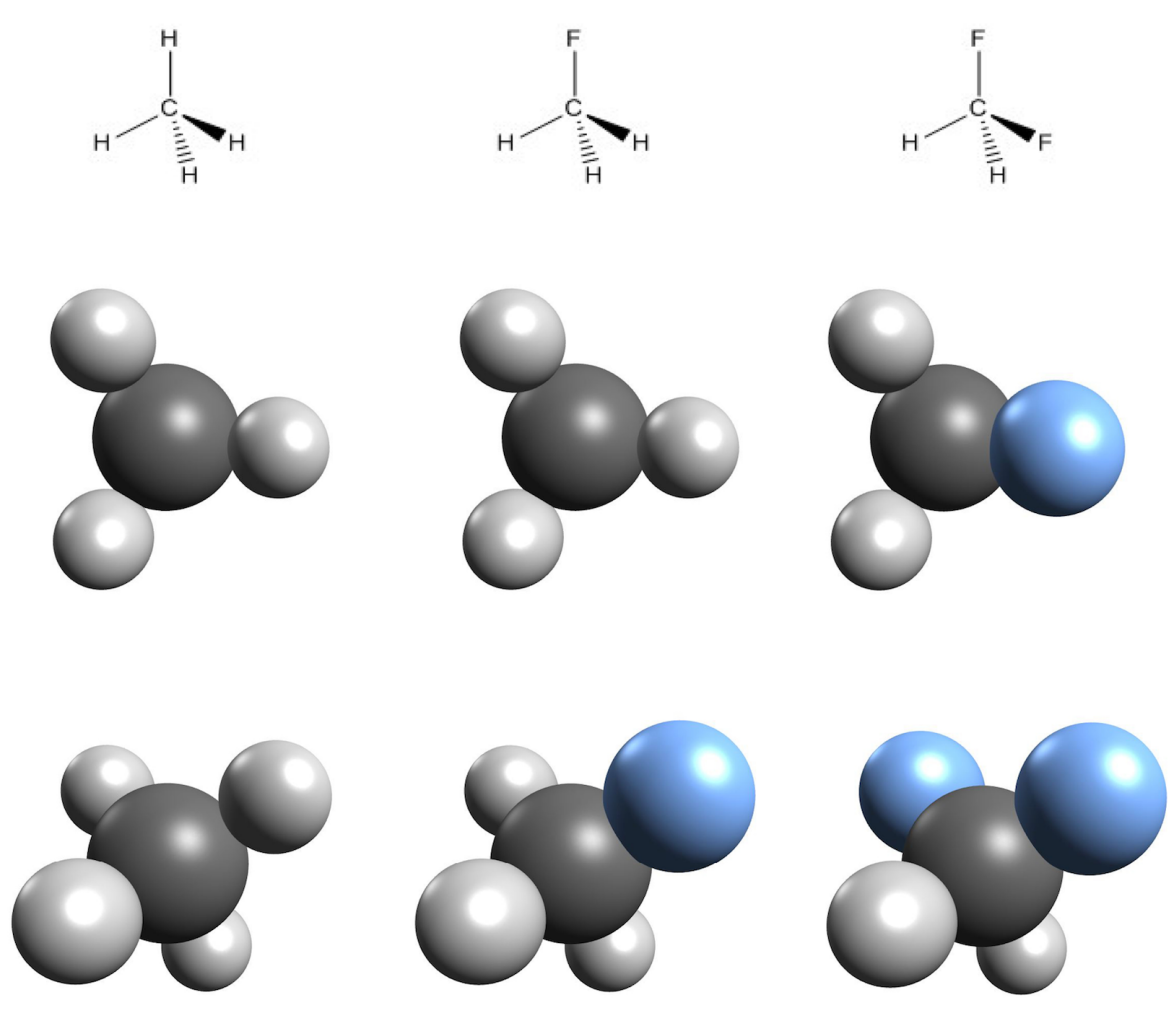
Methane, fluoromethane, and difluoromethane in Lewis representation (top, left to right), in ball and stick representation from a viewpoint covering one atom in each molecule (middle) and in ball and stick representation from a shifted viewpoint (bottom). Carbon = dark grey, hydrogen = light grey, fluorine = blue.

To illustrate the concept methane, fluoromethane, and difluoromethane are compared (Figure 1). If fluoromethane is orientated in a way that the observer’s point of view is along the carbon–fluorine bond, it appears indistinguishable from methane in the analogous orientation since the fluorine atom is hidden behind the carbon atom. Difluoromethane, however, cannot be viewed in a similar orientation; the second fluorine atom is always visible. Hence, only methane and fluoromethane are perspective isomorphs while difluoromethane is not.

**Figure 2:**
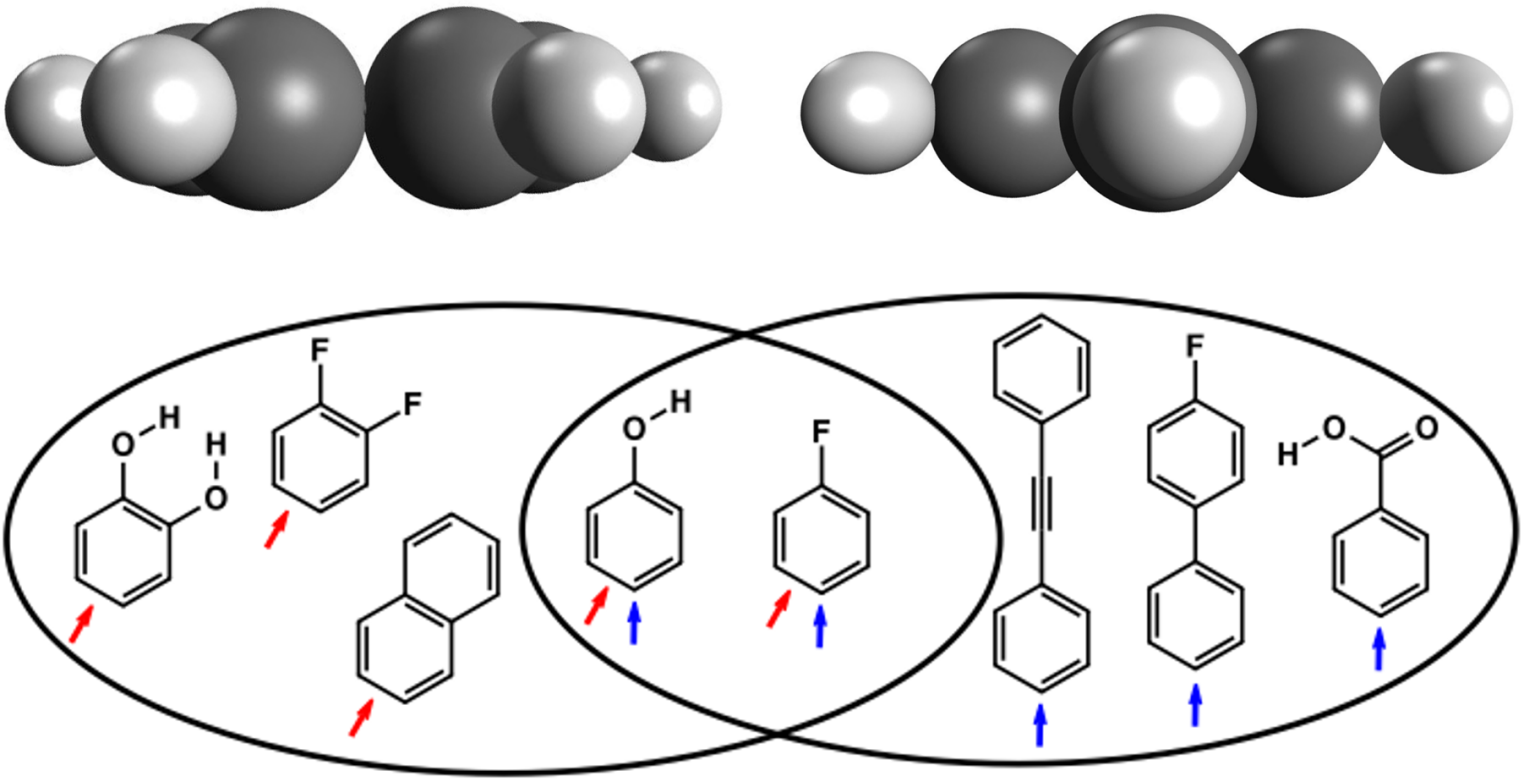
Multiple allocations of molecules to PI classes. Molecules that can be allocated to a group on the left: 1,2-dihydroxybenzene, 1,2-difluorobenzene, naphthalene. Molecules that can be allocated to a group on the right: diphenylacetylene, 4-fluorobiphenyl, benzoic acid. Molecules that can be allocated to both groups: benzene (not shown), phenol, fluorobenzene (the arrows in red and blue indicate the two different view points of the observer).

According to the viewpoint, one molecule can be assigned to different PI classes. In Figure 2 different aromatic compounds are shown. While 1,2-dihydroxybenzene is perspective isomorphic to 1,2-difluorobenzene or naphthalene, benzene and phenol can be grouped into the same PI class. These two compounds can, however, be placed into a different PI class, when viewed from a different angle. This way, they are perspective isomorphe with 4-fluorobiphenyl or benzoic acid.

### Nomenclature/coding of perspective isomorphs

In order to make the concept of perspective isomorphs practicable and unmistakably understandable the introduction of a new nomenclature is inevitable. For coding the different groups of perspective isomorphs we adapted the basic structure of the INCHI code (short for International Chemical Identifier) [16], yet we propose an alternate version called the PI code. The InChI code describes chemical substances by transforming their structural information into an explicit character sequence for identification. The information is given in terms of layers around the atoms and their bond connectivity, tautomeric information, isotope information, stereochemistry, and electronic charge information. In contrast, in the PI code only the visible atoms are indicated in the information about the chemical structure.

In addition, the letter X is introduced in order to replace a non-visible atom if needed. This definition is necessary, as the code must ensure that the encoded molecules maintain their specific arrangement in space, which is highly relevant for the concept of perspective isomorphs. Substitutes with a specific position within the bond are numbered consecutively with X_1_, X_2_, X_3_…X*_n_* , unspecific ones are indicated as a mere X and can stand for a variable number of atoms including 0. As a consequence, also the point of view is given by this information since the substitutes indicate the non-visible parts of the molecule, making an explicit mention of the point of view in the PI code redundant.

There is the possibility of introducing a sub-layer for the substitutes X*_n_* to precisely classify the molecules included by a PI code*.* This gives the option of not only providing information about a whole group of perspective isomorphs, but also about certain molecules belonging to this group. The layer x, for instance, defines the atoms of the substitutes X_1_-X*_n_*, layer hx indicates the hydrogen bonds of the substitues X_1_-X*_n_*. The information regarding the indications of the PI-group is separated from information about the specific perspective isomorph by the characters //.

For a more user-friendly understanding we suggest to refer to the dimensionality of the encoded molecule in a new layer that is positioned at the beginning of the PI code, directly after the numbering of the version code by using the characters 1D, 2D or 3D.

**Figure 3:**
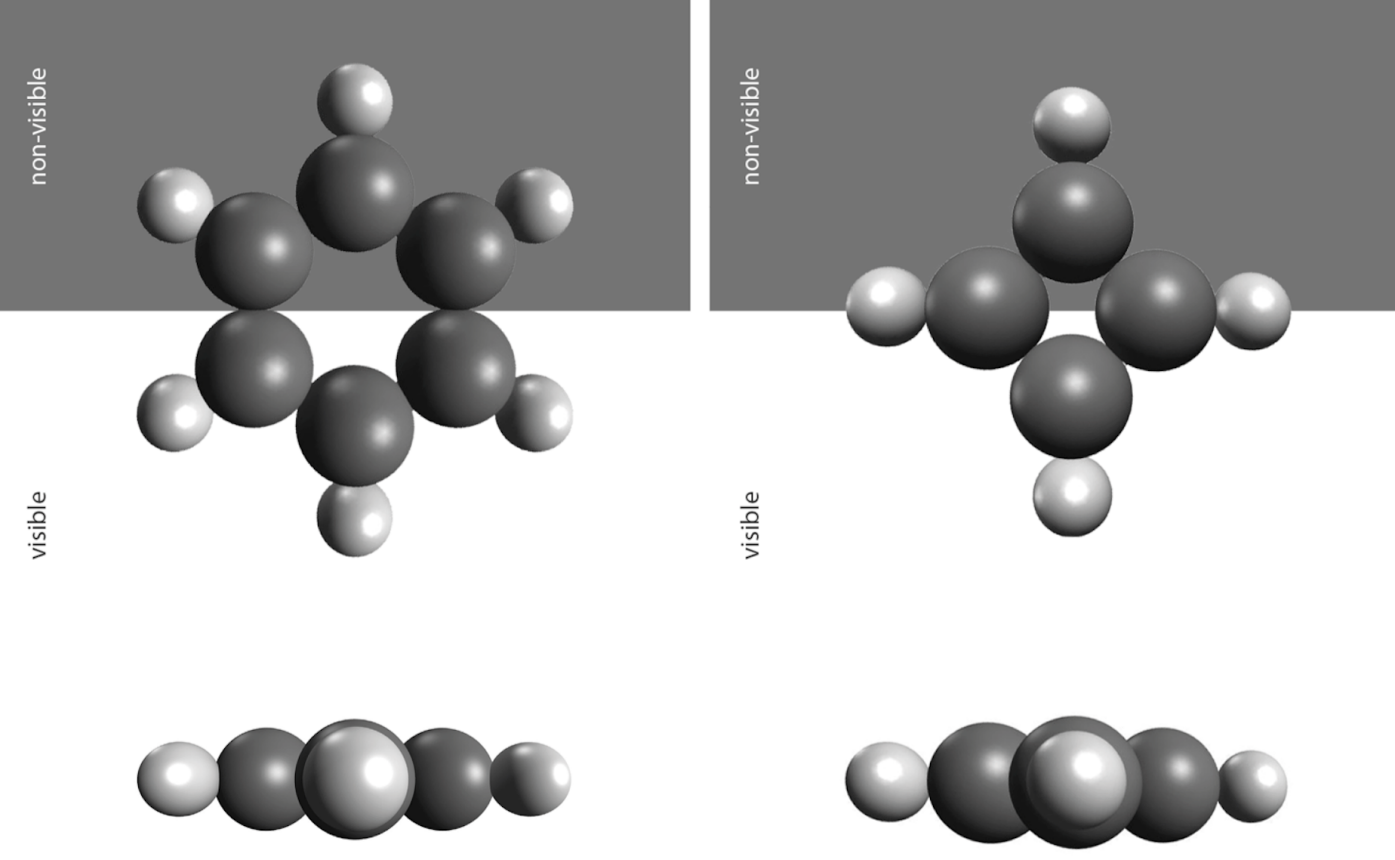
Left: benzene from two different angles, PI code of benzene: PI = 1/2D/C3H3/cX1-1-2-3-X2-X3-X1/h1-3H; PI code with specific indication of benzene: PI = 1/2D/C3H3/ cX1-1-2-3-X2-X3-X1/h1-3H//xX1-X3C/hxX1-X3H. Right: cyclobutadiene from two different angles, PI code of cyclobutadiene: PI = 1/2D/C3H3/cX1-1-2-3-X1/h1-3H; Pi code with specific indication of cyclobutadiene: PI = 1/2D/C3H3/cX1-1-2-3-X1/h1-3H//xX1C/hxX1H.’ IMPORTANT: no subscript in the PI-code.

The composition of the PI code is illustrated by the example of benzene and cyclobutadiene (Figure 3). The first number (1) states the version of the PI code. In case of later revisions, this number distinguishes an earlier from the current version. The next sequence describes the dimensionality; as benzene is a flat molecule, the value is 2D for two-dimensional. The molecular formula of the visible atoms (C_3_H_3_) follows after that. The subsequent sections in the PI code are started by a specific letter to designate the content since not all of these parts have to be present. The next section starts with the initiator ‘c’ describing the connection between the visible as well as the hidden atoms. In the case of benzene it is composed of three atoms X1 to X3, for which the visible atom 3 is connected to the first invisible atom X_1_ and the invisible atom X_3_ is connected to the visible atom 1 closing the six-membered benzene ring. In the next section, starting with an ‘h’ the connectivity in the visible part is given by stating that each atom 1 to 3 is connected to one H-atom. This description represents the general description of this PI class. In order to specify one specific sub-class or sub-member of a PI class, the hidden parts can be described in more detail. In this case the general PI code is continued after the ‘//’. In the x-section the hidden atoms X_1_ to X_3_ are assigned to be C-atoms. After that the hx-section defines that each of the X-atoms is connected to one H-atom. The PI code of cyclobutadiene is constructed in an analogous manner. However, in this case, there is only one atom in the hidden connection to account for the different geometry. Although this difference is barely observable the different angle slightly changes the distances in the visible arrangement.

### Artistic experiment and discussion

Apart from the mathematical definition of perspective isomorphs, it was consequently of interest to examine their arrangement and coverage in actual space. None of the common model representations of molecules turned out to be solely conclusive for our matter because they either do not allow the atoms any corporeality (Newman projection, Haworth projection, skeletal formula, structural formula, Fischer projection, Natta projection, wireframe) they do not represent them spherically shaped (van der Waals spheres, stick model) or they convey the idea of gaps between the individual atoms and leave out their decrease of inner density (ball and stick model, wire model). Due to the limited validity of each individual model and the recognition of the complementarity of the different model concepts, it was necessary to look for new ways of representing atoms. Artists are experts in new, innovative image creations. They have the ability to process various aspects and complex information in images.

**Figure 4:**
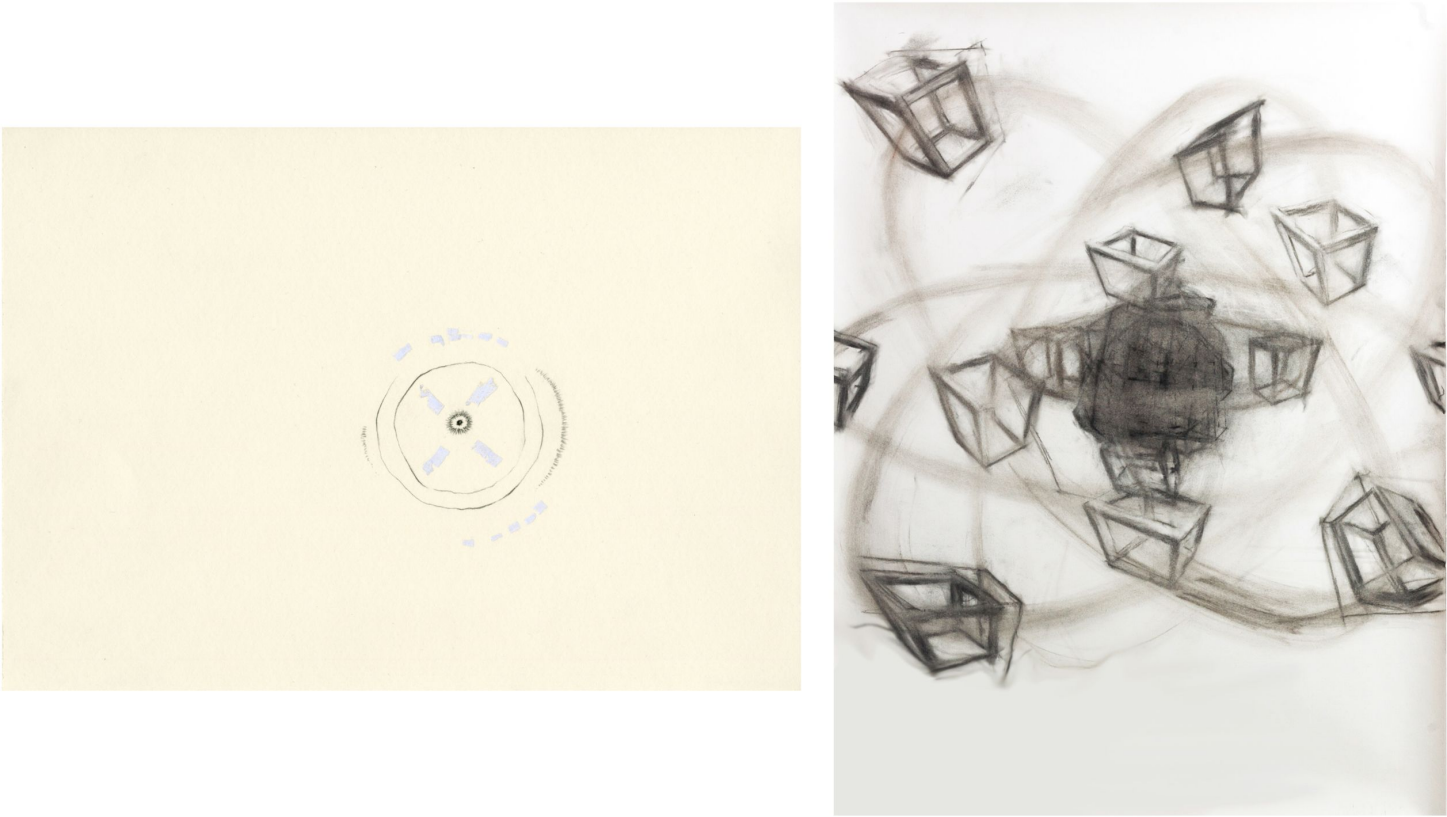
The atom carbon by Patrick Borchers (left) and Christoph Kern (right).

**Figure 5:**
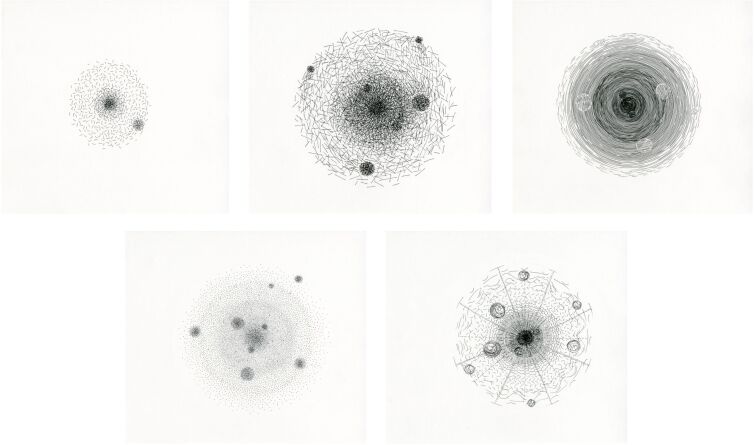
Artistic interpretation by Jette Flügge of the atoms hydrogen, carbon, nitrogen, oxygen, and fluorine (from top left to bottom right).

In order to alternate the predominant omniscient view and in recognition of the existing multiperspectivity of knowledge and systems of representation the three artists Patrick Borchers, Jette Flügge, and Christoph Kern, who treat spatiality in their artistic concepts in very different ways, were invited to participate in the project and to create graphic conceptions of the five considered elements (see Figure 4, Figure 5 and Supporting Information File 1 for details about the artists). This resulted in three very different graphic series of the five atoms. The graphics show individual interpretations of atomic structures and properties, but they remain recognizable and comprehensible within the framework of the specifications. The focus of the graphic interpretation of Kern was on the movement and dynamics within the atom (Figure 4, right). Borchers emphasized more the filigree, almost substance-less form of atoms Figure 4, left). Flügge interpreted the atoms each with very individual properties and structures (Figure 5). The resulting artistic drawings on the one hand represent works of art with a high intrinsic value. In this project, on the other hand, they serve two further purposes: they can complement the previous pictorial ideas of atoms as innovative models and thus enrich the discourse on models in chemistry. In addition, the graphical models serve as tools in the process of spatial determination of perspective isomorphs.

**Figure 6:**
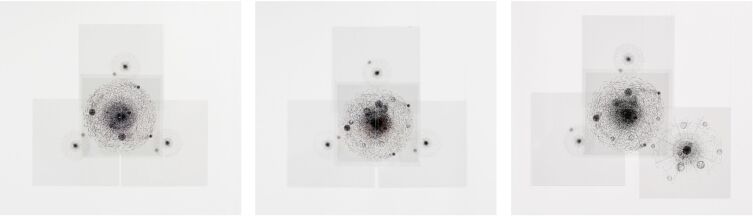
Hangings of methane (CH_4_), fluoromethane (CH_3_F), and difluoromethane (CH_2_F_2_) (from left to right) by Jette Flügge.

For this purpose, the artistic graphics were transferred via screen print in classical artistic technique onto transparent acrylic glass discs. The exemplary molecules (methane, fluoromethane and difluoromethane) were installed by hanging the individual atoms representing their conformation and the point of view to give the viewer the possibility to comprehend the congruency of the perspective isomorphs (Figure 6 and Supporting Information File 1). In doing so, it is crucially important that the artistic test setup allows the viewer more than a passive sensual experience due to its spatial installation. Following the radical constructivist Heinz von Foerster, only in the congruency between a sensation and an arbitrary change of position of the visual apparatus, the experiencing subject is able to generate a construction of the world [17]. Beyond the sensory organs, the whole body with its arbitrary movements is proven as a necessity in the visual faculty of spatiality with the artistic test setup, where it was impossible for the viewer to detect the isomorphic difference between several elements of a PI class without moving.

The classification of perspective isomorphic molecules could be effectively simulated with the installations created from the artists’ graphics. In an artistic and scientific exhibition in the Justus Liebig University of Giessen (August 9 – October 14, 2018) all artistic drawings were presented. In addition, three molecular models were installed based on the drawings printed on acrylic glass. From the marked point of view, methane (CH_4_) and fluoromethane (CH_3_F) could be considered as perspective isomorphic; difluoromethane (CH_2_F_2_) can easily be recognized to be non-perspective isomorphic to the other two. This visual experimental arrangement was installed in the course of the exhibition with all three artistic series (Flügge, Borchers, Kern). The exhibition was supplemented by a collection of molecular models and scientific posters (see Supporting Information File 1).

### Connection to modeling in drug discovery

The classification of compounds by shape has a long tradition in drug discovery [18–19]. With the development in computer technology powerful programs have been developed that allow comparing different structures and their affinity to potential binding sites [20]. However, in all approaches, again, the ‘viewing point’ is neglected. The researcher is present as an all-seeing being. Furthermore, the molecules are usually optimized in their geometry and conformation to the lowest energy before comparison. Our concept of *perspective isomorphs* is in this context unique: First, it is not restricted to a specific conformation due to an external requirement. Second, the hypothetical view from a specific point is compared, not the overall shape, placing crucial importance on the virtual observer in the process of analysis. Although this might appear as an artificial construct the contact of the investigated molecules with another entity with nanometer dimensions can be regarded as a congruent event. Although current software packages do not allow the automated classification of molecules in PI classes, the conceptual approach offers a novel way of categorizing for specific applications in medicinal chemistry, pharmacology or even in material sciences [21].

## Conclusion

With the proposal of perspective isomorphs, the transdisciplinary employment of artistic methods supports innovations in the field of chemical modeling. With this approach, molecules can be sorted by a new classification system offering a complementary strategy to compare structures at the atomic level. For this purpose, a definition of perspective isomorphs including a nomenclature system has been developed. The concept was translated into the field of human perception by involving the three artists Jette Flügge, Patrick Borchers, and Christoph Kern for creating representations of the atoms hydrogen, carbon, nitrogen, oxygen, and fluorine. The installation of molecules based on these graphics allowed the characterization of perspective isomorph classes. In general, this cross-disciplinary collaboration has introduced a meta-discourse about the nature and the diverse ways of cognition of matter beyond art and chemistry.

## Supporting Information

File 1 Additional example of perspective isomers with PI-code. Artist information for the project "Art in Chemistry"; curricula vitae of the artists; drawings by Patrick Borchers, Jette Flügge and Christoph Kern; hangings of CH_4_, CH_3_F and CH_2_F_2_ with atoms of Patrick Borchers and Christoph Kern, impressions from the exhibition.
