# Supplementary material for: Perspective isomorphs – a new classification of molecular structures based on artistic and chemical concepts
Source: Beilstein J Org Chem. 2019 Sep 30;15:2319–26. doi: 10.3762/bjoc.15.224 (PMC6808190; doi:10.3762/bjoc.15.224)
Supplement: File 1 — Additional example of perspective isomers with PI-code. Artist information for the project "Art in Chemistry"; curricula vitae of the artists; drawings by Patrick Borchers, Jette Flügge and Christoph Kern; hangings of CH4, CH3F and CH2F2 with atoms of Patrick Borchers and Christoph Kern, impressions from the exhibition. [file Beilstein_J_Org_Chem-15-2319-s001.pdf]

BEILSTEIN JOURNAL OF ORGANIC CHEMISTRY

Supporting Information

for

Perspective isomorphs – a new classification of molecular structures based on artistic and chemical concepts

Jannis Neumann, Ansgar Schnurr and Hermann A. Wegner

Beilstein J. Org. Chem. **2019,** *15,* 2319–2326. doi:10.3762/bjoc.15.224

Additional example of perspective isomers with PI-code. Artist information for the project "Art in Chemistry"; curricula vitae of the artists; drawings by Patrick Borchers, Jette Flügge and Christoph Kern; hangings of CH_4, CH_3F and CH_2F_2 with atoms of Patrick Borchers and Christoph Kern, impressions from the exhibition

License and Terms: This is a supporting information file under the terms of the Creative Commons Attribution License (http://creativecommons.org/licenses/by/4.0). Please note that the reuse, redistribution and reproduction in particular requires that the authors and source are credited.
The license is subject to the *Beilstein Journal of Organic Chemistry* terms and conditions: (https://www.beilstein-journals.org/bjoc)

Content:

1. Additional example of perspective isomers with PI-code: S2
2. Artist information for the project "Art in Chemistry" S3
3. Curricula vitae of the artists: S4
4. Drawings by Patrick Borchers…………………………………………………………………..S7
5. Drawings by Jette Flügge ……………………………………………………………..………S10
6. Drawings by Christoph Kern……………………………………………………………….……S13
7. Hangings of CH_4, CH_3F and CH_2F_2 with atoms of Patrick Borchers…………………………...S16
8. Hangings of CH_4, CH_3F and CH_2F_2 with atoms of Christoph Kern……………………………..S18
9. Project-Exhibition at the Justus-Liebig-University Giessen……………………………………..S20

1. Additional example of perspective isomers with PI-code

Cis-1,3-diflourocyclohexane *vs.* fluorocyclohexane:

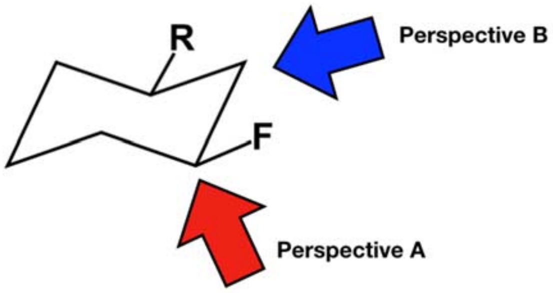

Two different perspectives for *cis*-1,3-diflourocyclohexane (R = F) *vs*. fluorocyclohexane (R = H).

Perspective A

cis-1,3-diflourocyclohexane:

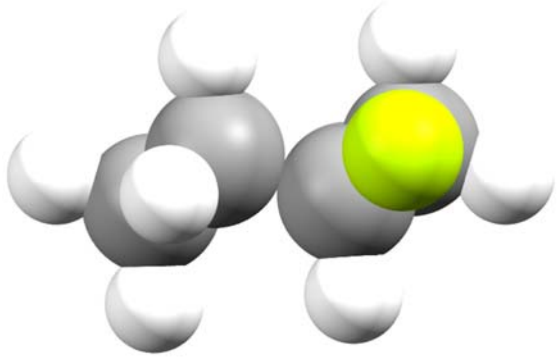

PI=1/3D/C4H7F/cX1-1-2-4(5)3-X2-X1/h4H,1-3H2/t3-/m0/s1

Fluorocyclohexane:

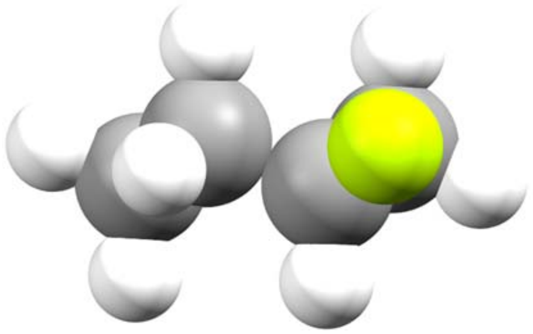

PI=1/3D/C4H7F/cX1-1-2-4(5)3-X2-X1/h4H,1-3H2/t3-/m0/s1

Perspective B

cis-1,3-diflourocyclohexane:

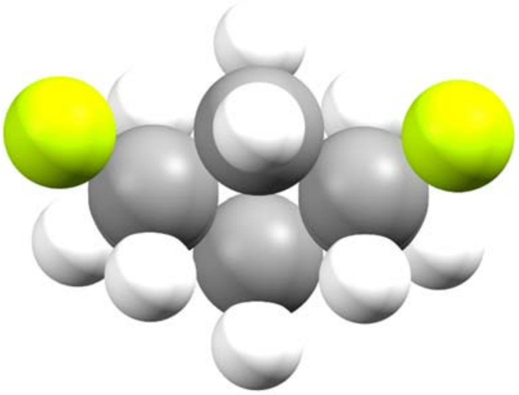

PI=1/3D/C4H9F2/cX1-3(5)1-4(6)X2-2-X1/h2-4H,1,X1-X2H2/ t3-,6+

Fluorocyclohexane:

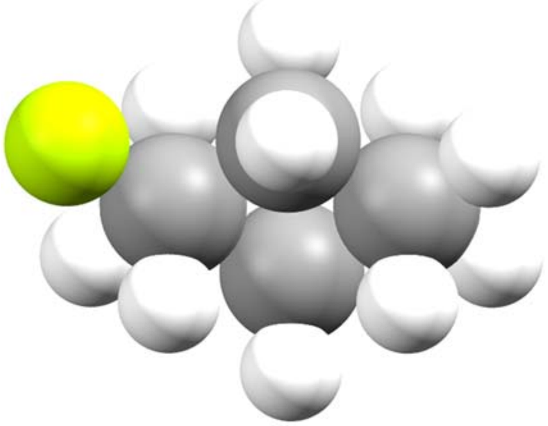

PI=1/3D/C4H10F/cX1-4(5)1-2-X2-3-X1/h3-4H,1-2,X1-X2H2/ t1-/m0/s1

2. Artist information for the project "Art in Chemistry"

For the project "Art in Chemistry" free graphic interpretations for the elements hydrogen, carbon, nitrogen, oxygen and fluorine are to be found. Current models of chemistry are only models and none can claim to represent reality. Accordingly, the artistic interpretation can / should be detached from previous representations. Nevertheless, there are a few, limited guidelines to follow, which are derived from the theory behind the project. Instead of a single solution, five different graphical representations are also to be chosen, which specifically pick up the properties of the five elements. In which form this is realized remains up to the artist.

General requirements: One atom of the elements hydrogen, carbon, nitrogen, oxygen and fluorine should be displayed in one color. A distinction of the various elements is made solely by the graphical track. The individual atoms have all a spherical shape, which has a higher density in the middle and decreases concentrically towards the outside. This represents the different probable locations of the electrons. The electrons are permanently in motion within the atom. The size of the atoms is arbitrary, it should be noted that all elements are about the same size, with the exception of hydrogen, which is roughly half the size of the other atoms.

Specifics for the individual elements:

1. Hydrogen - 1 external electron and roughly half the size of the other elements
2. Carbon - 4 external electrons and 2 internal electrons
3. Nitrogen - 5 external electrons and 2 internal electrons
4. Oxygen - 6 external electrons and 2 internal electrons
5. Fluorine - 7 external electrons and 2 internal electrons

3. Curricula vitae of the artists

Patrick Borchers

German artist. Born 1975 in Herdecke / Germany;

1998-2003 studies in Art-Education and Fine Arts, Technical University Dortmund. Since 2006 curator for various exhibitions; teaching assignments at various institutions in Düsseldorf, Dortmund, Essen. 2010 Artist in Residence in Linz / Austria, 2012 Artist in Residence in Berlin; since 2014 artistic assistant for graphics at the University of Dortmund.

http://www.patrickborchers.de

Selected Projects and Publications:

2018 Publication: Patrick Borchers –Luftlinien, Kerber Verlag

2017 „blumengießen“, Schlachthaus, Gießen

2016 engagée#3 –desire, in: é | engagée–Magazin für politisch-philosophische Einmischungen, Wien

2015 engagée#1 –Unruhe bewahren, Graphics on protest in: é | engagée, Magazin für politisch-philosophische Einmischungen, Wien

2011 Publication: protestmelody–Patrick Borchers, hyperzineverlag

2010 Urban Interventions, Linz, Österreich

Awards:

2011 Kunstpreis Ennepe-Ruhr, Galerie des Kreishauses Schwelm, (Cat.)

2008 Eurode-Kunstpreis 2008, Forum für Kunst und Kultur Herzogenrath

2007 DEW21 Kunstpreis 2007, Dortmund

2005 Strom_Linie_Form, Stadtmuseum Hattingen, (Cat.)

Jette Flügge

German Artist; 1974 born in Wickede/Ruhr;

2004-2009, Studies in Graphics and Sculpture at the Free Academy if Fine Arts Essen; 2010-2017 Studies of Art-Education at the Technical University of Dortmund, 2013 Bachelor ofArts, 2017 Master of Education.

2013-2017 teaching assignments for graphics in Dortmund, Essen, Giessen. 2017-2018 head of the workshop of graphics at the University of Giessen, since 2018 Technical University of Dortmund.

http://www.jette-fluegge.de

Exhibitions (Selection):

2018 „Landschaft", FKF-Projekt, Städtische Galerie Iserlohn

2017 „Draußen im Reihenhaus. Kunst bei Kallus", Bonn, (E) & K. Heyltjes

2017 „bevorzugte Gebiete", blam! Produzentengalerie, Dortmund (E)

2017 „Zuflurstücke", Städtische Galerie Iserlohn (E)

2017 „Augenscheinlichkeiten2", Studierende von Prof. Bettina van Haaren, Dortmunder U (K)

2015-2017 „Druckreif", Xylon Deutschland: StäditischesKunstmuseum SpendhausReutlingen, Die Drostei Pinneberg, Städtische Galerie und Kunstverein Speyer, Kloster Bentlage Rheine (K)

2016 „bevorzugte Gebiete", blam! Produzentengalerie, Dortmund (E)

2016 „Zuflurstücke", Städtische Galerie Iserlohn (E)

2015-2017 „Revolution", Kunstraum am Rhein DCKD, Düsseldorf (K)

Awards:

2014 3.Offenes Atelier am Rothaarsteig, Brilon Auswahl

2014 Förderpreis der Märkischen Bank, Hagen

2014 2.Preis der XVII. Deutschen Int. Grafik-Triennale Frechen

2012 Kunstpreis der TU Dortmund für Graphik

Christoph Kern

German artist. Born 1960 in Munich / Germany.

1979-81 Studies in Philosophy und History, 1981-88 Studies in Painting and Graphics at the Academy of Fine Arts Munich.
Since 1994 teaching assignments for painting at the FH Potsdam, Universities of Paderborn, Greifswald, Giessen. 1997 Postgraduate scholarship under the terms of an agreement between the United States Information Agency (USIA) in Washington, D.C. and the State of Bavaria; 1999 – 2018 Guestprofessor for Painting and Media at the University of Paderborn, since 2019 Guestprofessor for Painting at the Justus-Liebig-University of Giessen.

https://www.christophkern.net

Individual Exhibitions (Selection)

2018 Chimaera. CAT, Tavira, Portugal

2017 Rochade, Galerie Heufelder, München

2017 Papierarbeiten, werkschau.galerie, München

2016 The Uncertainty of the Painted Cube, mute gallery, Lisbon

2014 CubicWorldsrev., VKU Berlin2013 Open Space, Uni Paderborn

2011 CubicWorldsII Galerie Michael Heufelder, München

2009 standsforattitude, locusluxgallery, brussels2008Flachland, Galerie Heufelder, München

2008 CubicWorlds, Filipp RosbachGalerie, Baumwollspinnerei Leipzig (Katalog)

2008 The Diversity of the Painted Cube, Titanium Yiayiannos Gallery, Athen (Katalog)

2007/2002 Galerie Ulysses, Wien;

4. Drawings by Patrick Borchers

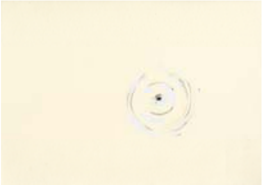
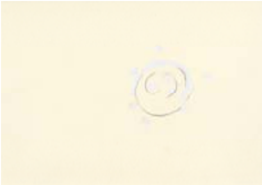
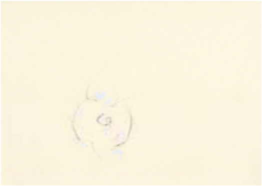

Series of five Drawings, each 21x29,9 cm, Graphite Pencil and TipEx on Paper, 2018

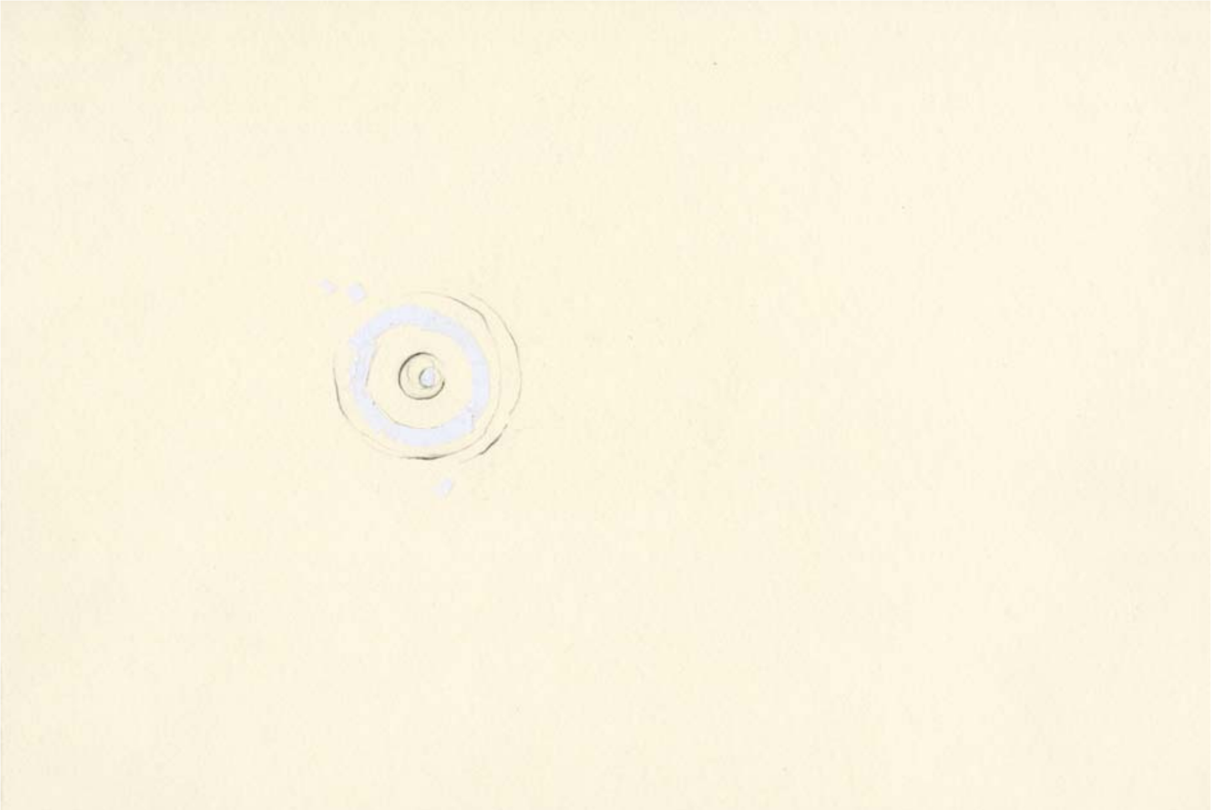

Hydrogen

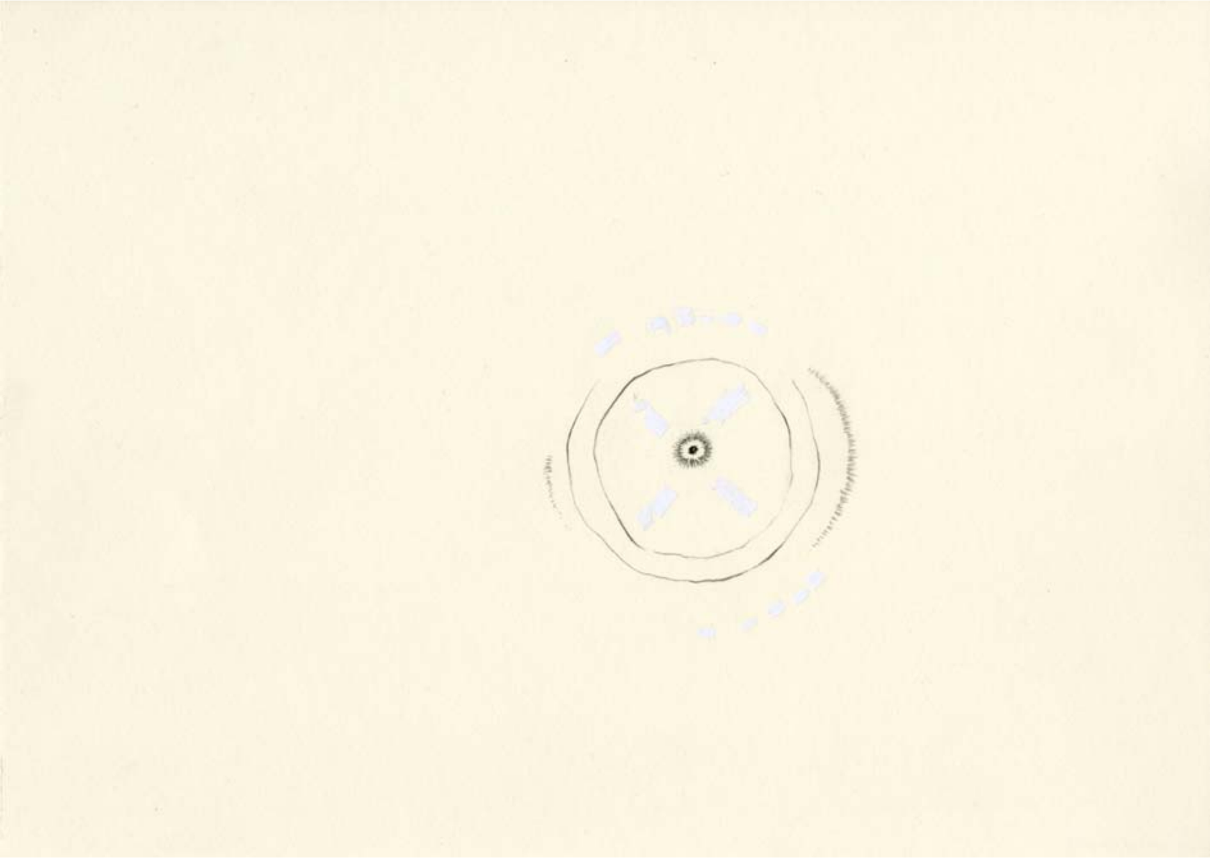

Carbon

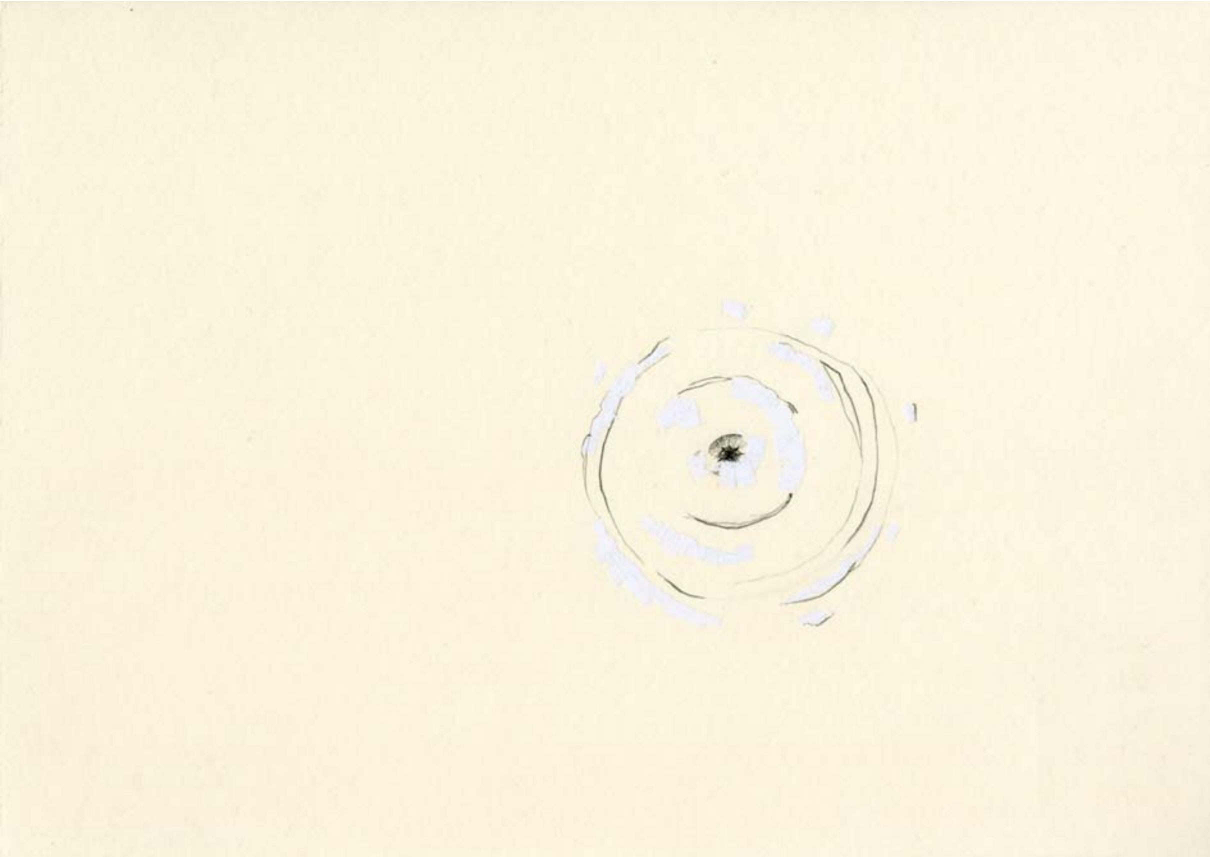

Nitrogen

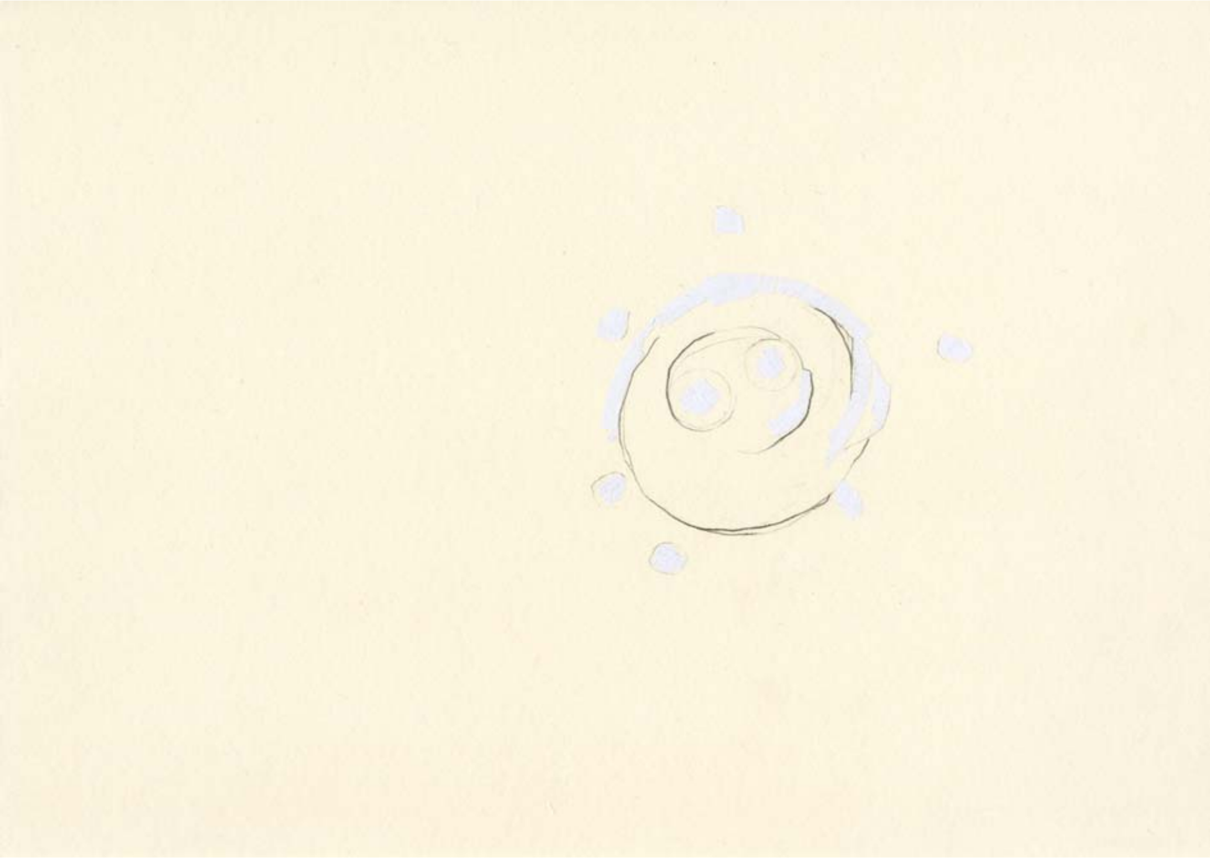

Oxygen

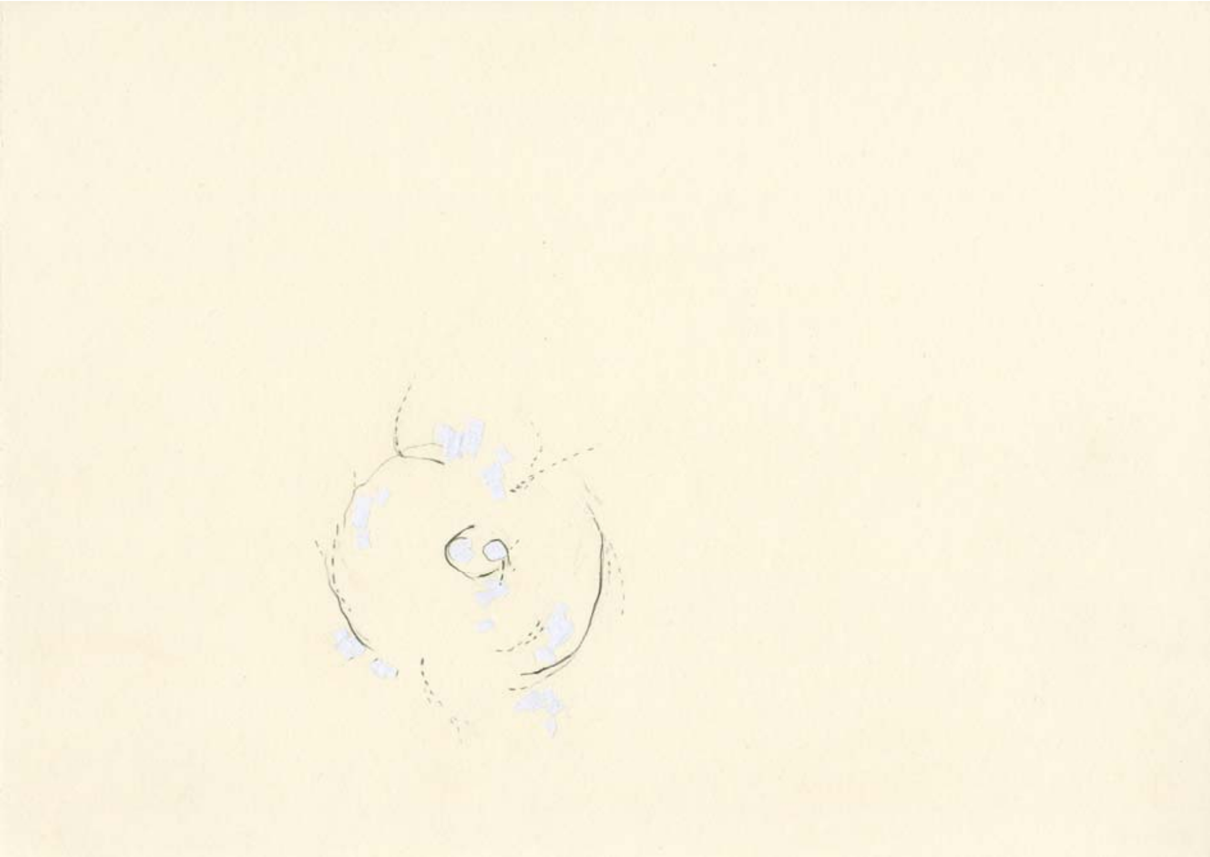

Fluorine

5. Drawings by Jette Flügge

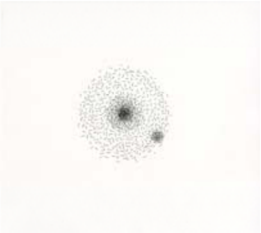
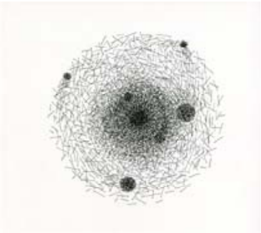
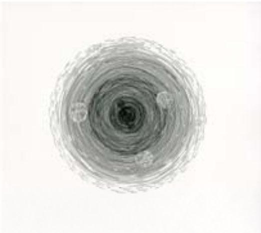
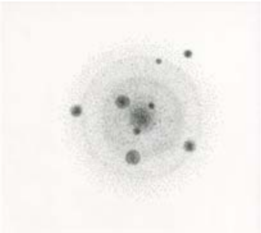
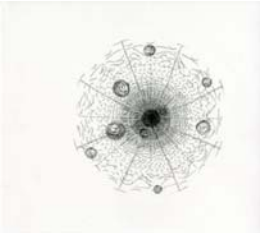

Series of five Drawings, each 26,5x29,7 cm, Fineliner on Paper, 2018

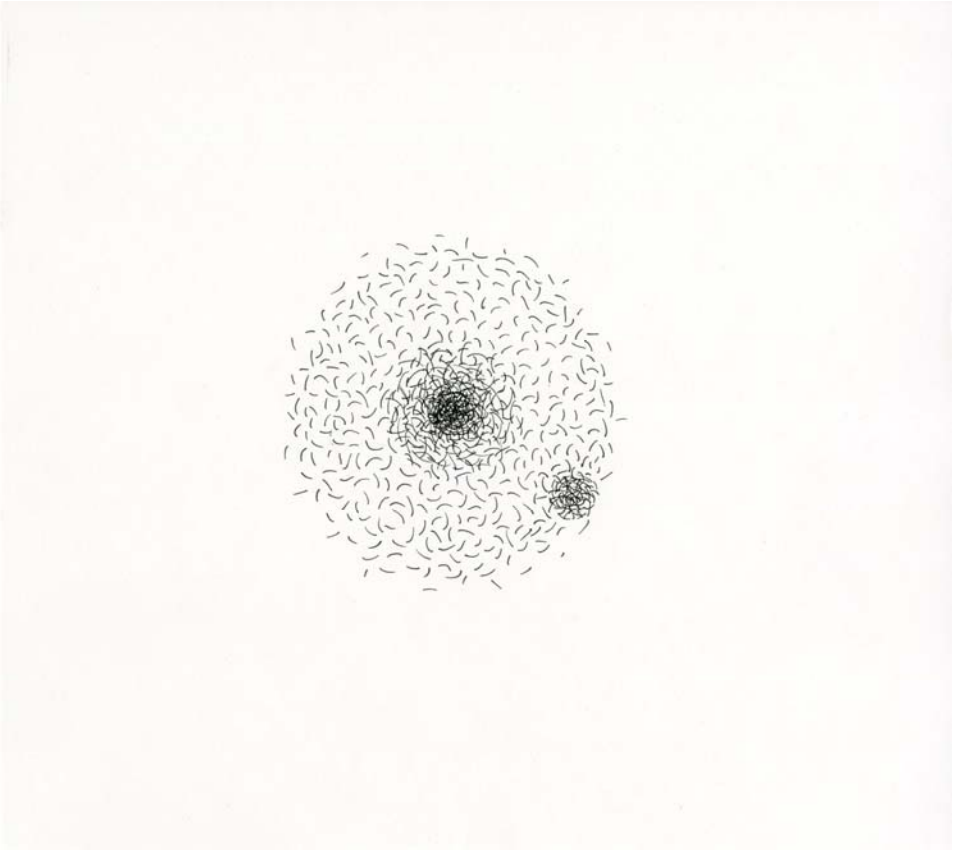

Hydrogen

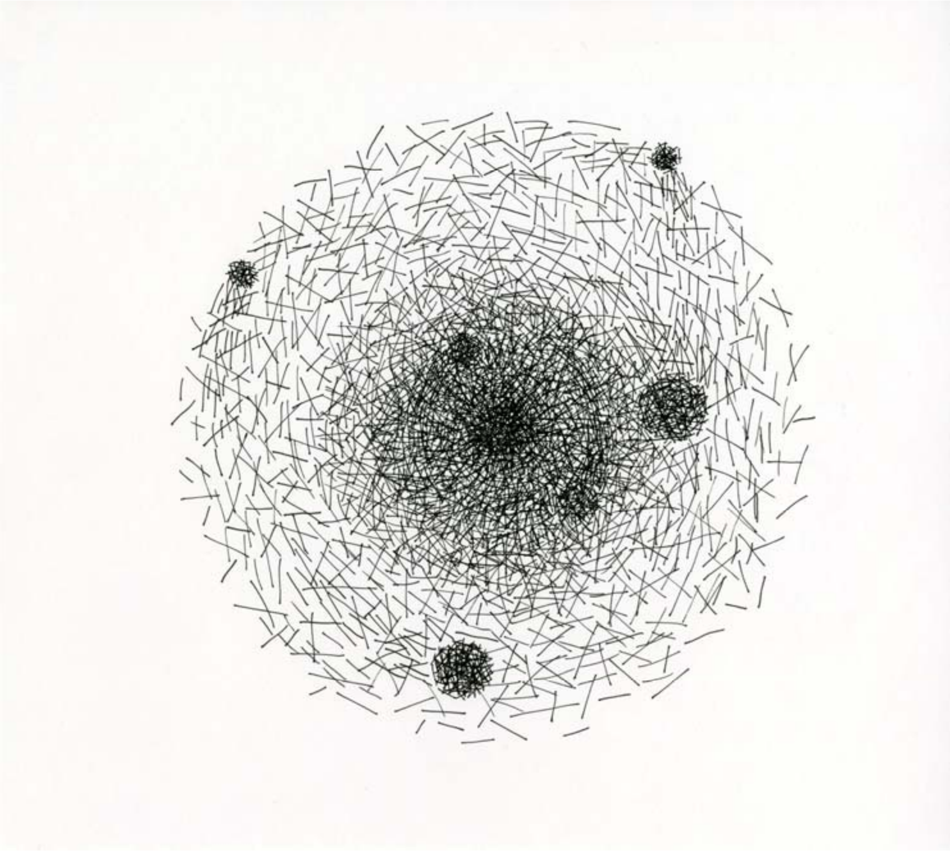

Carbon

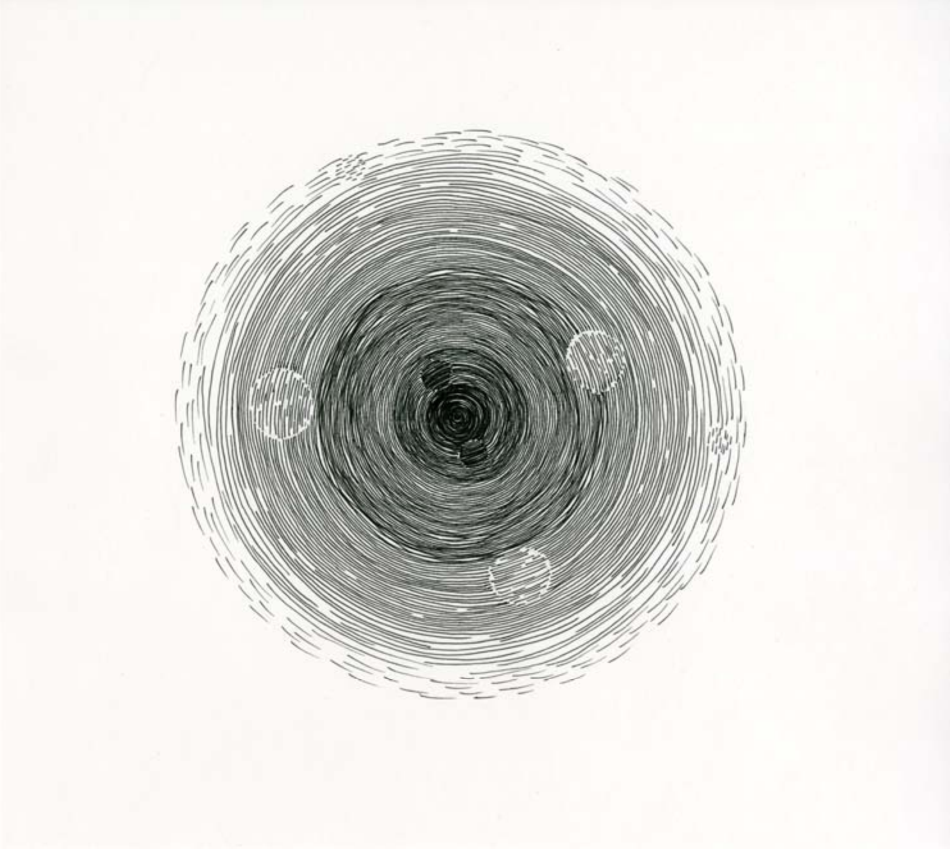

Nitrogen

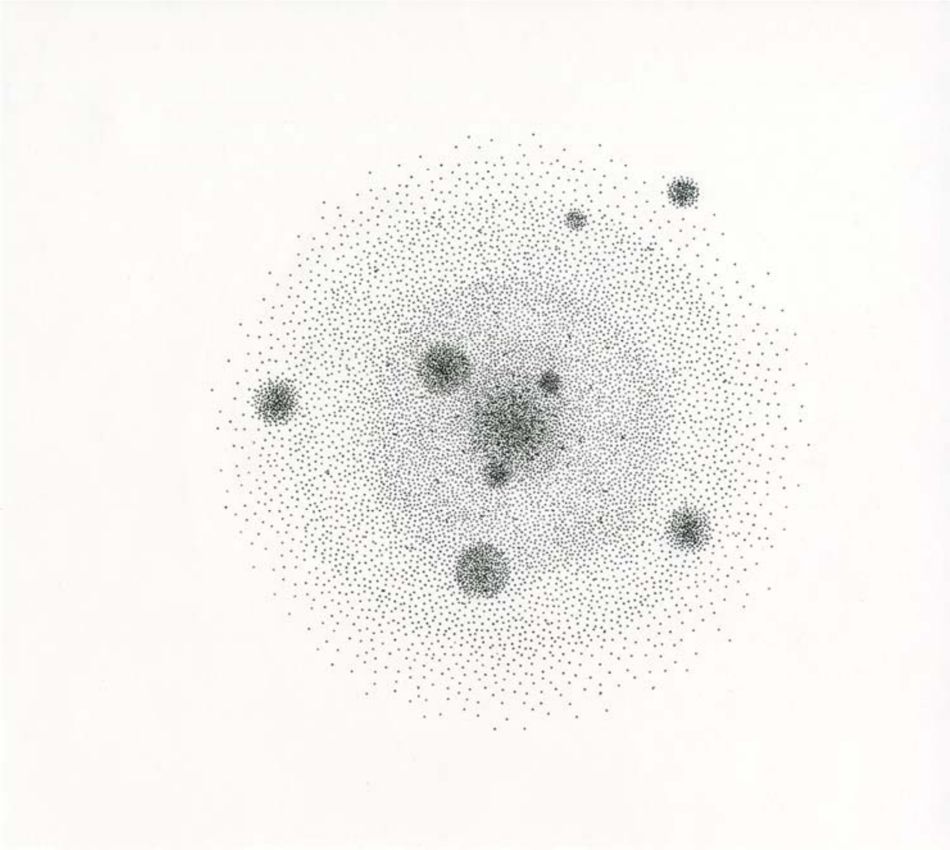

Oxyugen

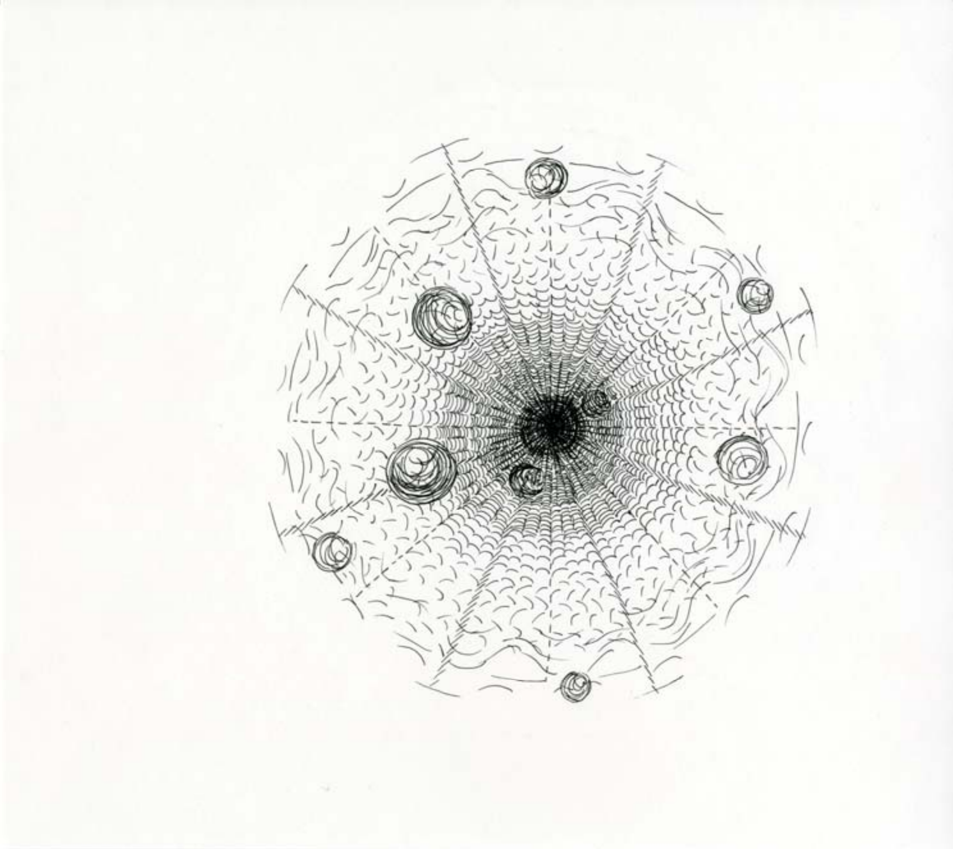

Fluorine

6. Drawings by Christoph Kern

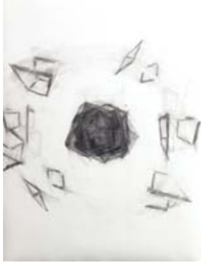
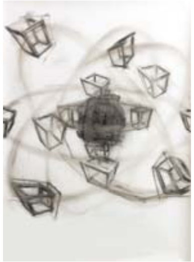
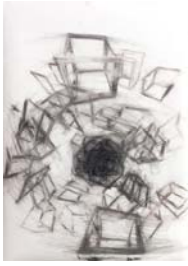
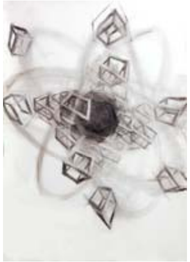
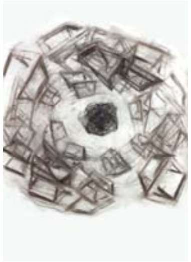

Series of five Drawings, each 107x76 cm, Charcoal on Paper, 2018

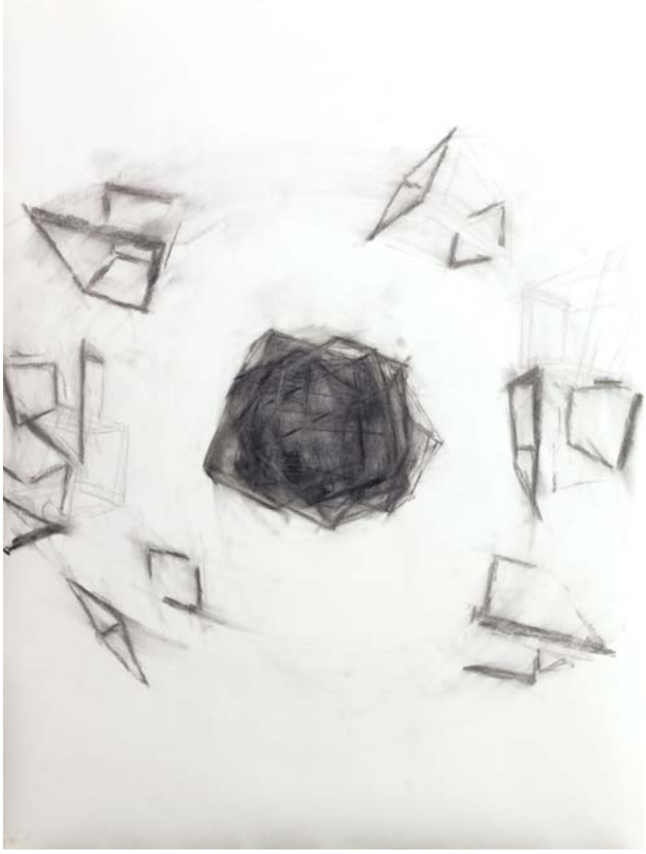

Hydrogen

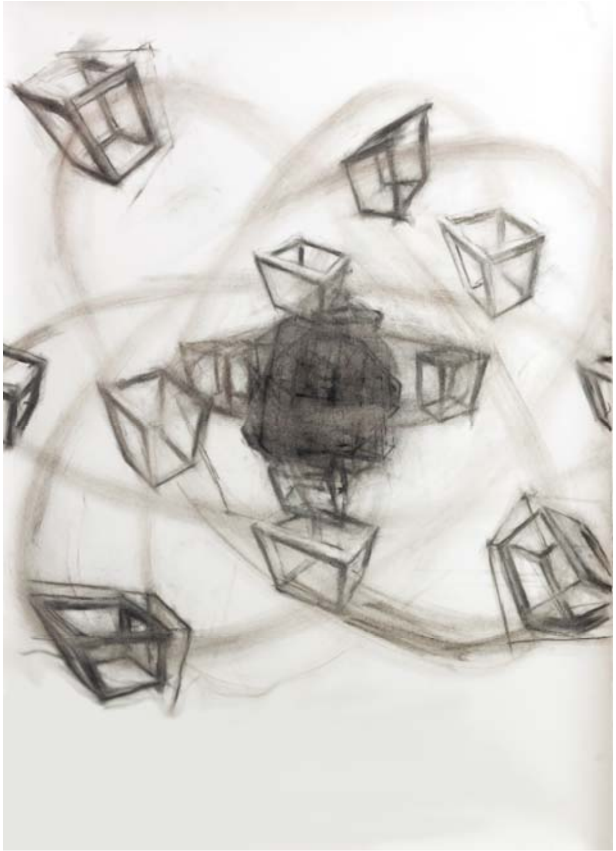

Carbon

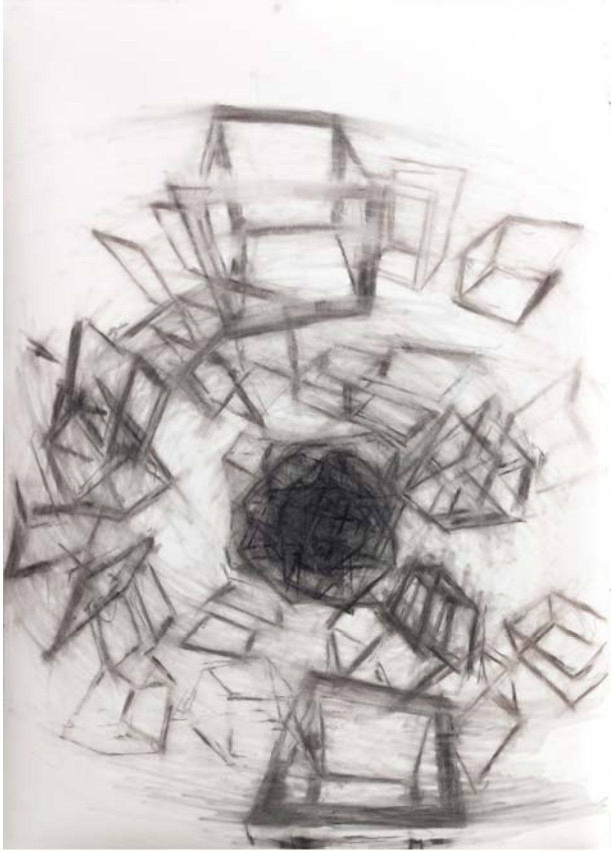

Nitrogen

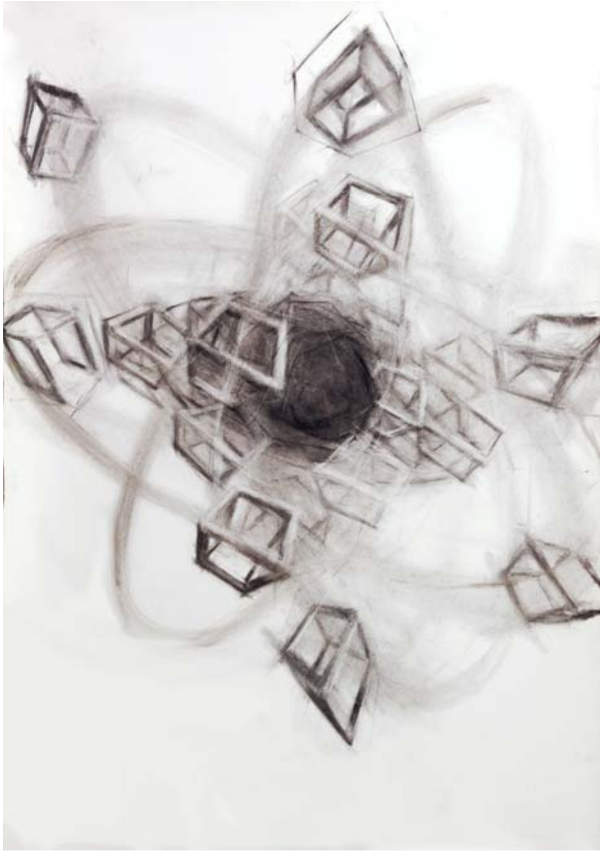

Oxyugen

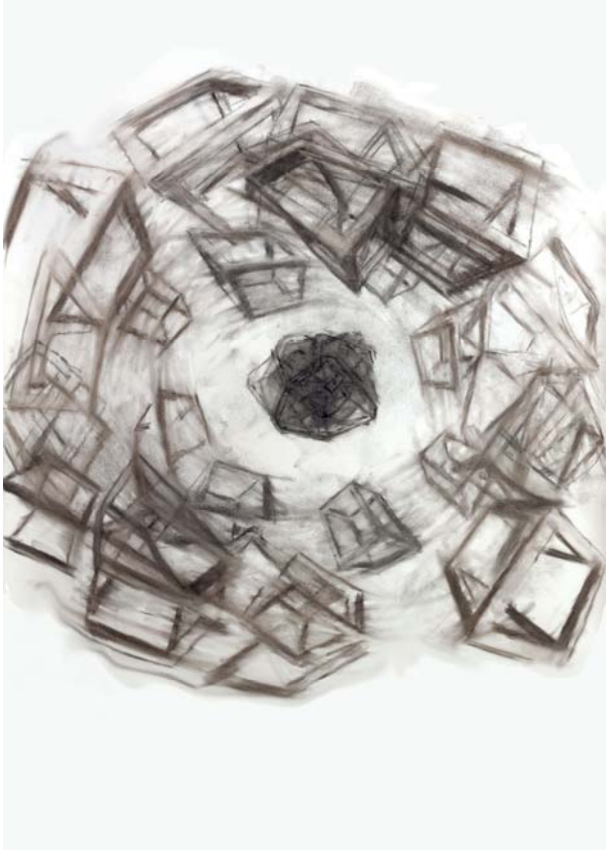

Fluorine

7. Hangings of CH_4, CH_3F and CH_2F_2 with atoms of Patrick Borchers

Installation of hanging Screenprints of artistic drawings, printed on Acrylic Glass

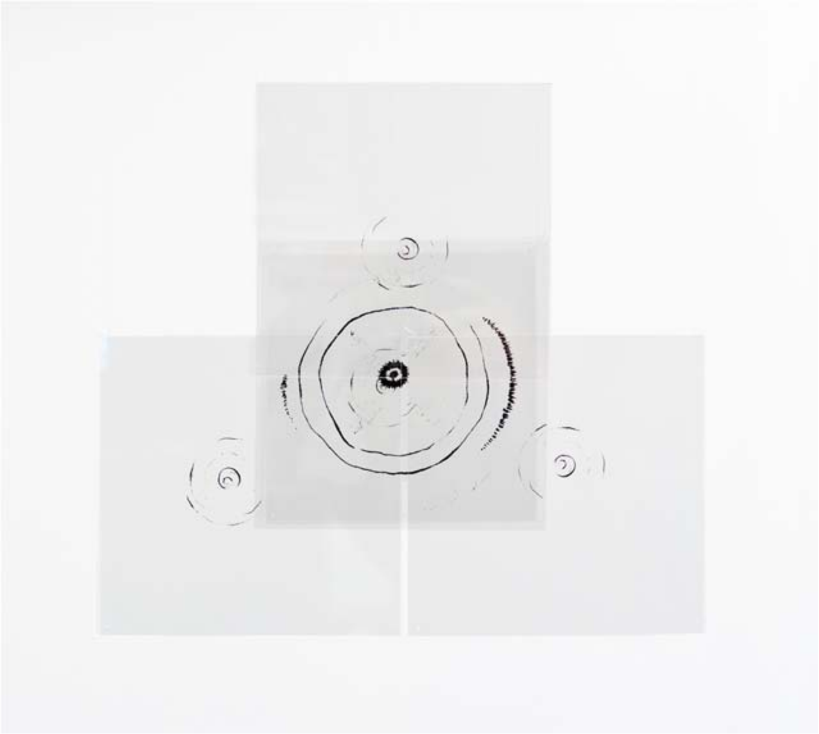

Methane

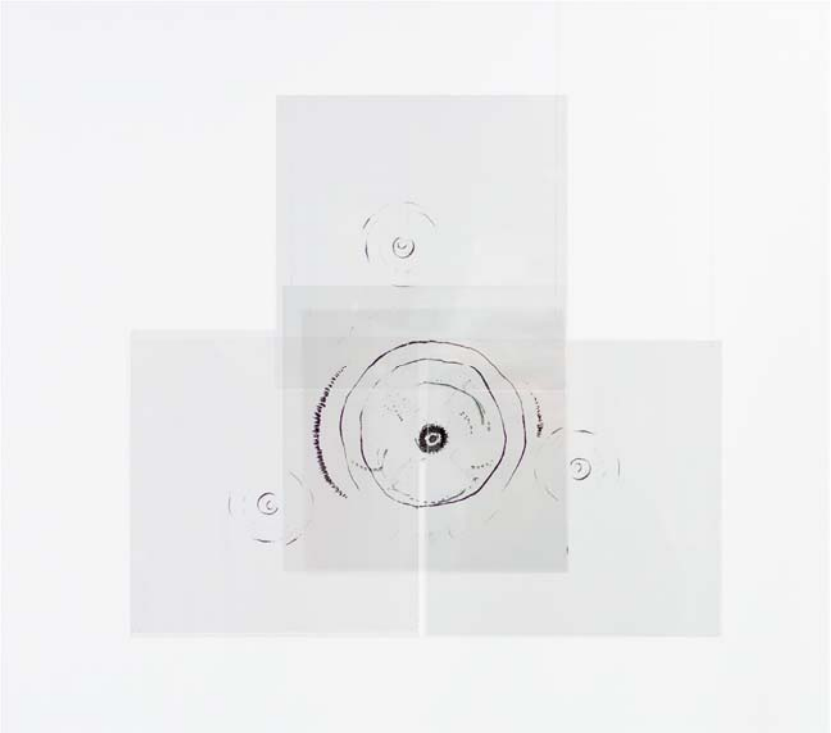

Fluoromethane

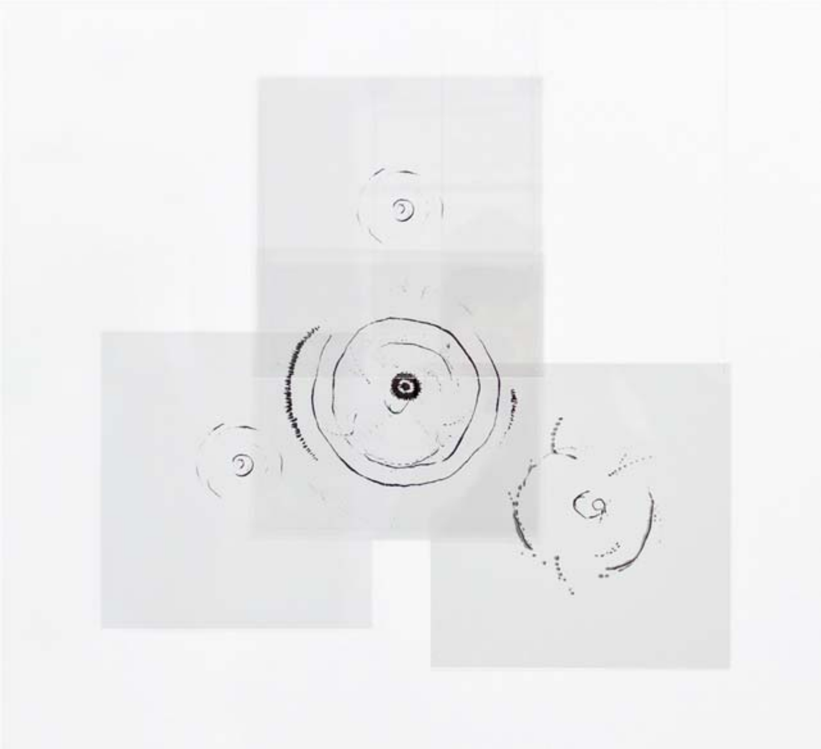

Difluoromethane

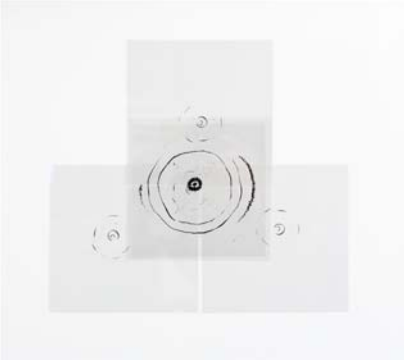

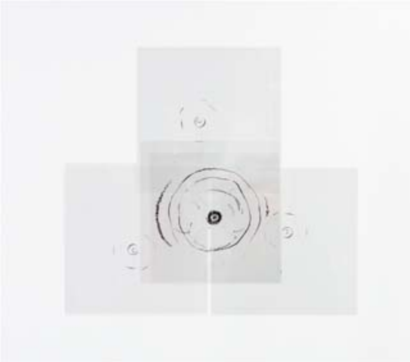

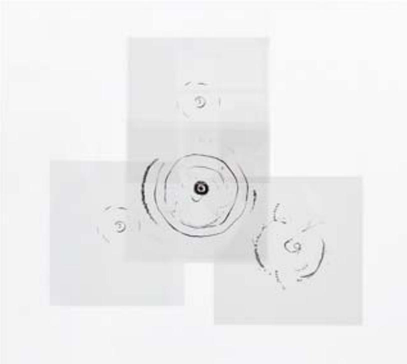

Methane – Fluoromethane - Difluoromethane

8. Hangings of CH_4, CH_3F and CH_2F_2 with atoms of Christoph Kern

Installation of hanging Screenprints of artistic drawings, printed on Acrylic Glass

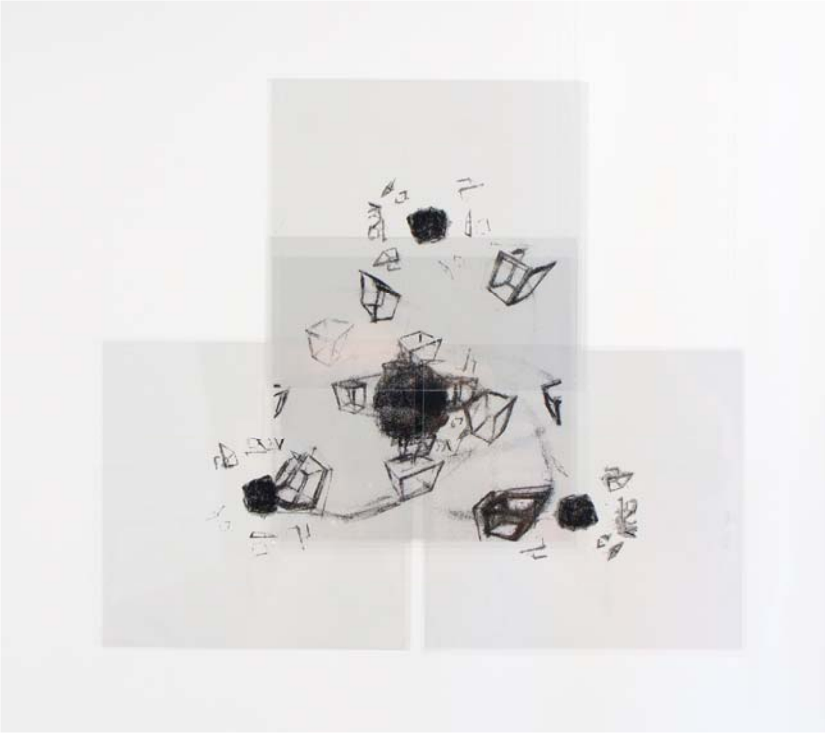

Methane

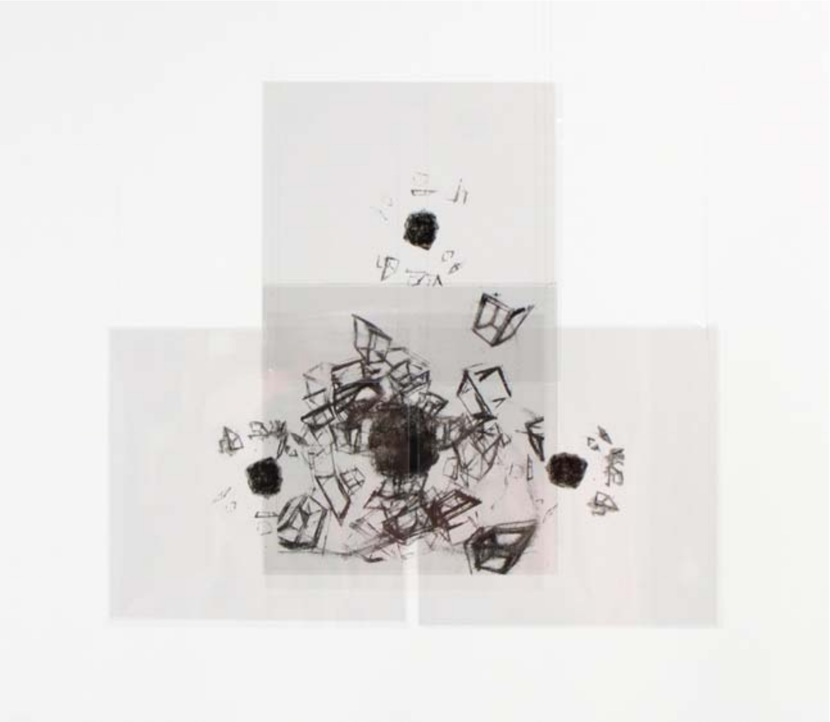

Fluoromethane

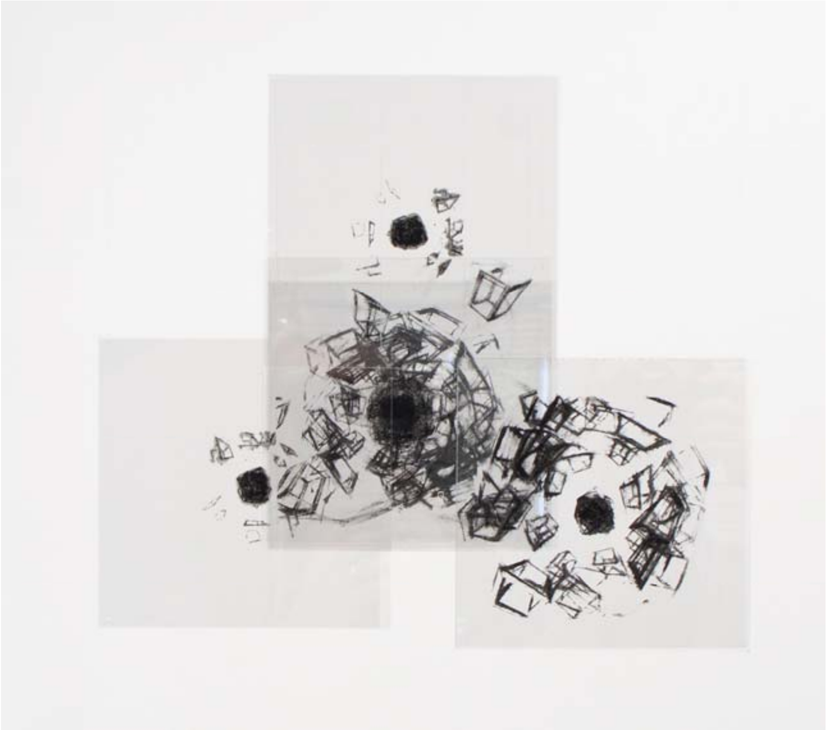

Difluoromethane

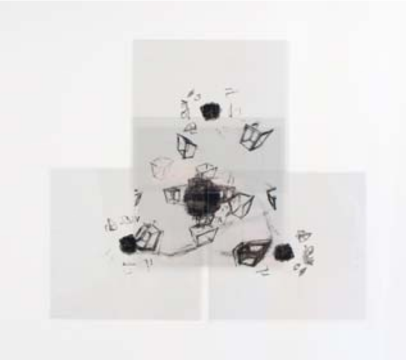

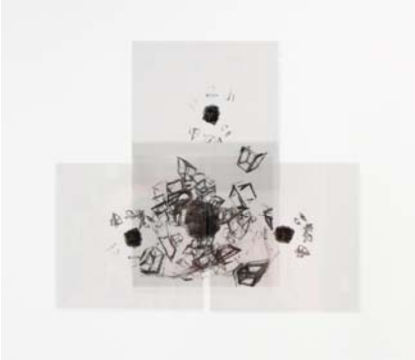

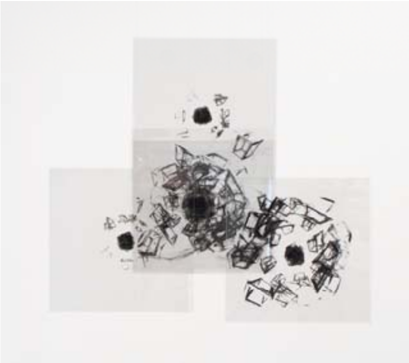

Methane – Fluoromethane – Difluoromethane

9. Project-Exhibition at the Justus-Liebig-University Giessen, 2018

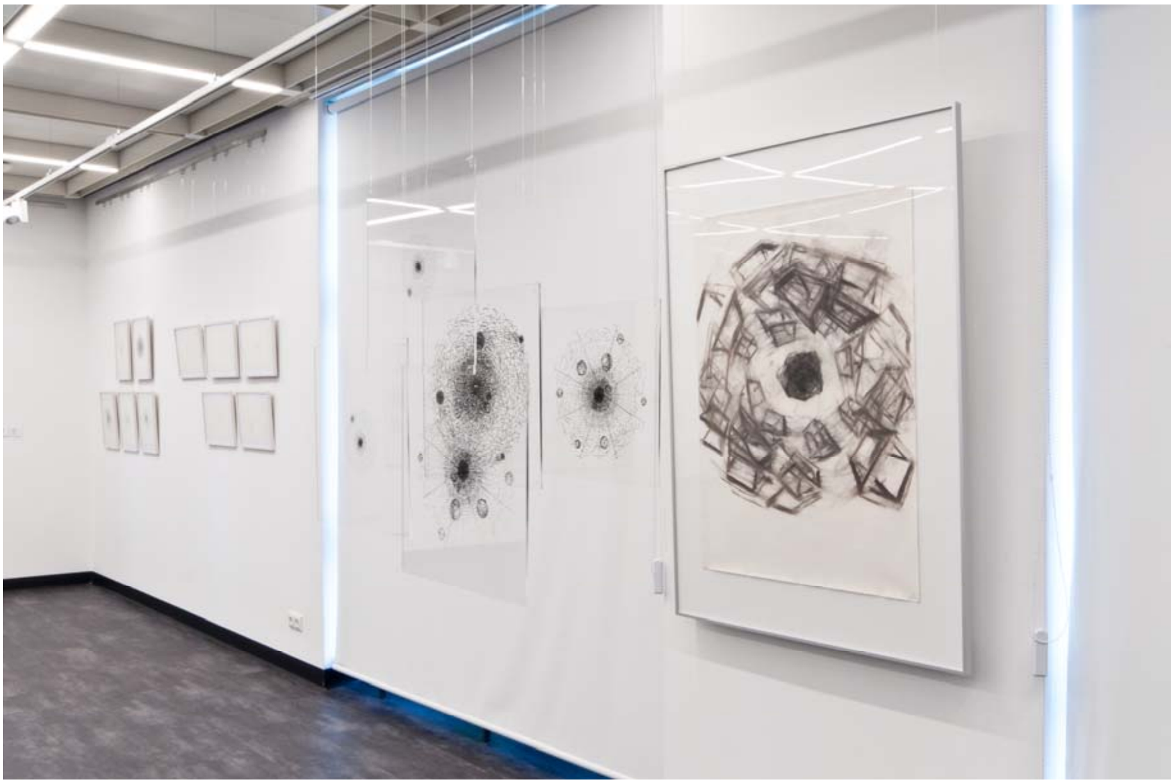

Exibition View, Justus-Liebig-University Giessen, 2018

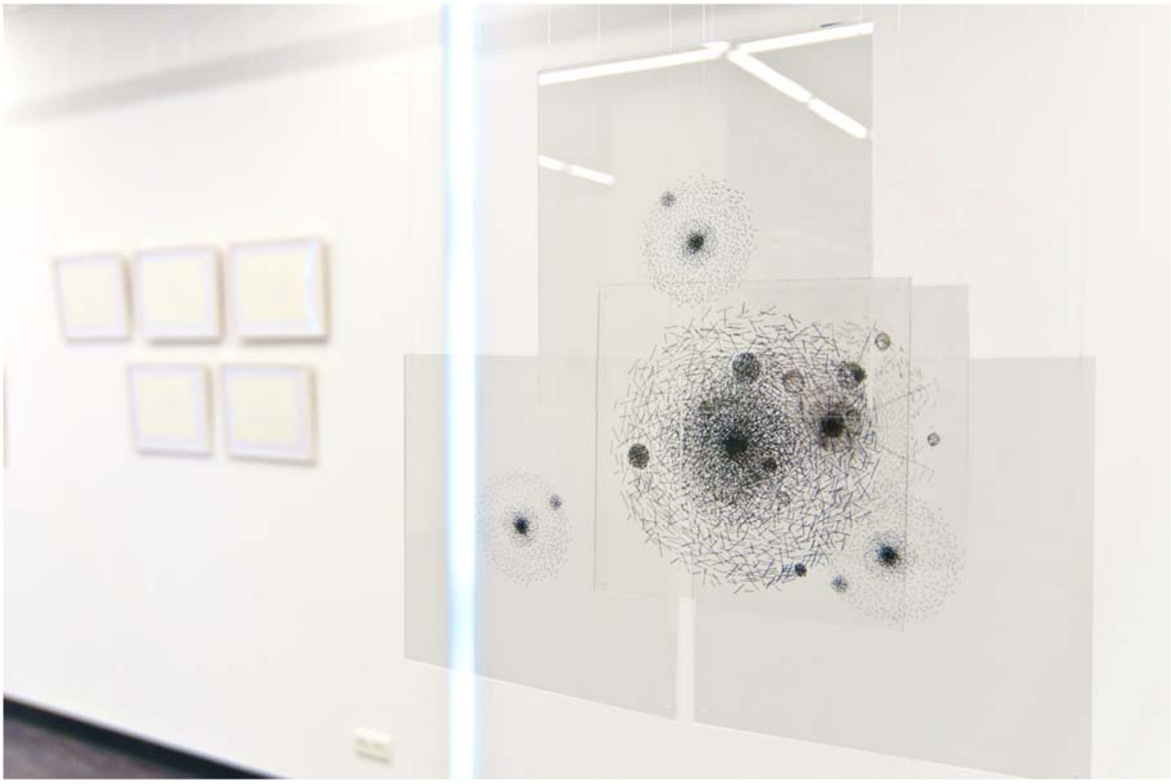

Installation View, hanging of Jette Flügges drawings, Justus-Liebig-University Giessen, 2018
